# Application of metabolomics in intrahepatic cholestasis of pregnancy: a systematic review

**DOI:** 10.1186/s40001-022-00802-z

**Published:** 2022-09-14

**Authors:** Zhuoqiao Yang, Mengxin Yao, Chunhua Zhang, Xuan Hu, Yi Zhong, Xiangxiang Xu, Jieyun Yin

**Affiliations:** 1 Department of Epidemiology and Health Statistics, Medical College of Soochow University, Suzhou, China; 2 Department of Obstetrics, Gusu School, The Affiliated Suzhou Hospital of Nanjing Medical University, Suzhou Municipal Hospital, Nanjing Medical University, Suzhou, Jiangsu China; 3 School of Public Health, Medical College of Soochow University, 199 Renai Road, Suzhou, Jiangsu China

**Keywords:** Intrahepatic cholestasis of pregnancy, Metabolomics, Metabolites, Bile Acids

## Abstract

**Background:**

Intrahepatic cholestasis of pregnancy (ICP) is a severe idiopathic disorder of bile metabolism; however, the etiology and pathogenesis of ICP remain unclear.

**Aims:**

This study comprehensively reviewed metabolomics studies related to ICP, to help in identifying the pathophysiological changes of ICP and evaluating the potential application of metabolomics in its diagnosis.

**Methods:**

Relevant articles were searched through 2 online databases (PubMed and Web of Science) from January 2000 to March 2022. The metabolites involved were systematically examined and compared. Pathway analysis was conducted through the online software MetaboAnalyst 5.0.

**Results:**

A total of 14 papers reporting 212 metabolites were included in this study. There were several highly reported metabolites: bile acids, such as glycocholic acid, taurochenodeoxycholic acid, taurocholic acid, tauroursodeoxycholic acid, and glycochenodeoxycholic acid. Dysregulation of metabolic pathways involved bile acid metabolism and lipid metabolism. Metabolites related to lipid metabolism include phosphatidylcholine, phosphorylcholine, phosphatidylserine, sphingomyelin, and ceramide.

**Conclusions:**

This study provides a systematic review of metabolomics of ICP and deepens our understanding of the etiology of ICP.

## Introduction

Intrahepatic cholestasis of pregnancy (ICP) is a severe pregnancy complication, affecting 0.1–2% of pregnant women [1]. ICP is clinically characterized by pruritus and increased bile acids, and the symptoms usually disappear after labor [2]. Although essentially non-threatening from a maternal perspective, ICP is associated with an elevated risk of adverse fetal outcomes, including spontaneous preterm labor, meconium staining of the amniotic fluid, asphyxial events, and sudden intrauterine death [3, 4]. Despite that studies have shown that ICP is influenced by genetic predisposition, hormone levels, altered immunity, underlying liver disease, and environmental factors [5–7], the etiology and pathogenesis of ICP remain unclear. Meanwhile, the diagnosis of ICP mainly relies on detecting the serum concentration of total bile acid (TBA), but there are still several limitations [8]. For example, not all ICP patients have elevated TBA levels [9], and other liver diseases may also cause an increase in TBA [10]. Thus, enhanced research on the etiology, pathogenesis and diagnosis method is urgently required.

Metabolomics is a newly developed technology that can quantitatively analyze all metabolites in organisms and uncover the relative relationship between metabolites with physiological and pathological changes. Accordingly, it enables us to recognize the metabolites and metabolic pathways related to ICP, which could promote a deeper understanding of its etiology and pathophysiology, as well as boost its early prevention, diagnosis, and treatment.

In this study, we reviewed all metabolomics studies conducted on ICP over the last 20 years and systematically collected and analyzed the information from these researches. We summarized the significant changes in metabolic biomarkers and pathways of ICP to help us 1) understand the etiology and pathogenesis of ICP and 2) evaluate the potential application of metabolomics in ICP diagnosis.

## Methods

### Literature search

We obtained relevant publications from PubMed and Web of Science databases from January 2000 to March 2022, with the following searching terms: (“metabolome” or “metabolomics” or “metabolite” or “metabonomics” or “metabolic profiling” or “metabolic signature” or “metabolic biomarker” or “metabolic profile” or “metabolic portraits”) AND (“intrahepatic cholestasis of pregnancy”). All articles were searched and examined by two authors independently to assess their suitability for inclusion in the review, and a third researcher made a final decision in cases of disagreement.

### Inclusion and exclusion criteria

The inclusion criteria were (1) metabolomics studies on ICP, (2) full text in English, and (3) studies recording the positive or negative relationship between metabolite markers and ICP. The exclusion criteria were as follows: (1) review articles, (2) animal and cell studies, and (3) studies evaluating drug effects.

### Data extraction

We extracted the following information after reading the full articles and supplementary materials: (1) basic information of included studies, including first author, published date, and journal; (2) basic information of subjects, including sample size and singleton/twin; (3) study design, ICP diagnostic criteria, biological specimen, sampling time, and analytic platform; (4) the significant metabolites with changing trends. In addition, studies by the same first or corresponding author were checked whether there were overlaps in content.

### Statistical analysis

The frequencies on biological specimens, targeted/untargeted, analytic platforms, sample sizes, and frequently reported biomarkers were computed and charted. Pathway enrichment analysis and topology analysis were performed by the MetaboAnalyst 5.0 online software (https://www.metaboanalyst.ca) [11].

## Results

### Study characteristics

**Fig. 1:**
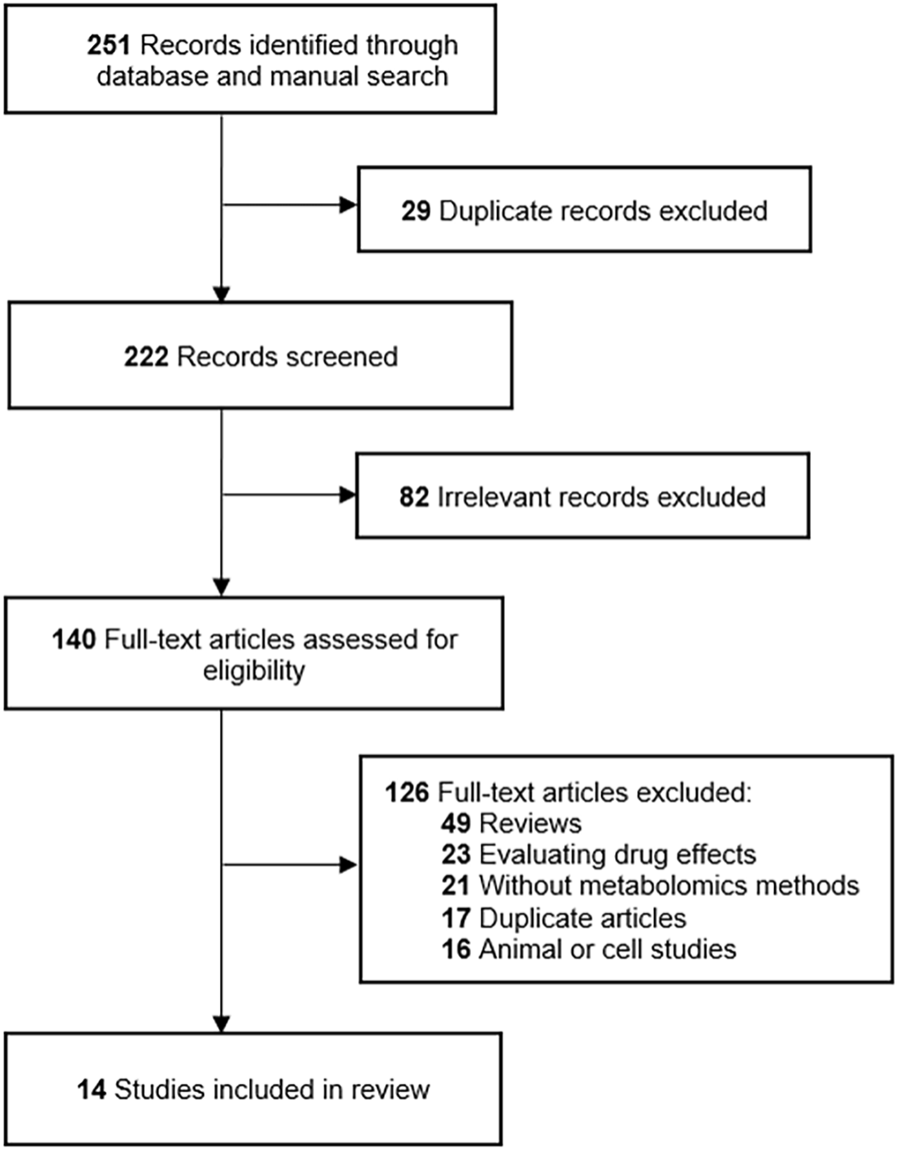
Flow diagram of literature search and study selection for metabolite markers of ICP

**Fig. 2:**
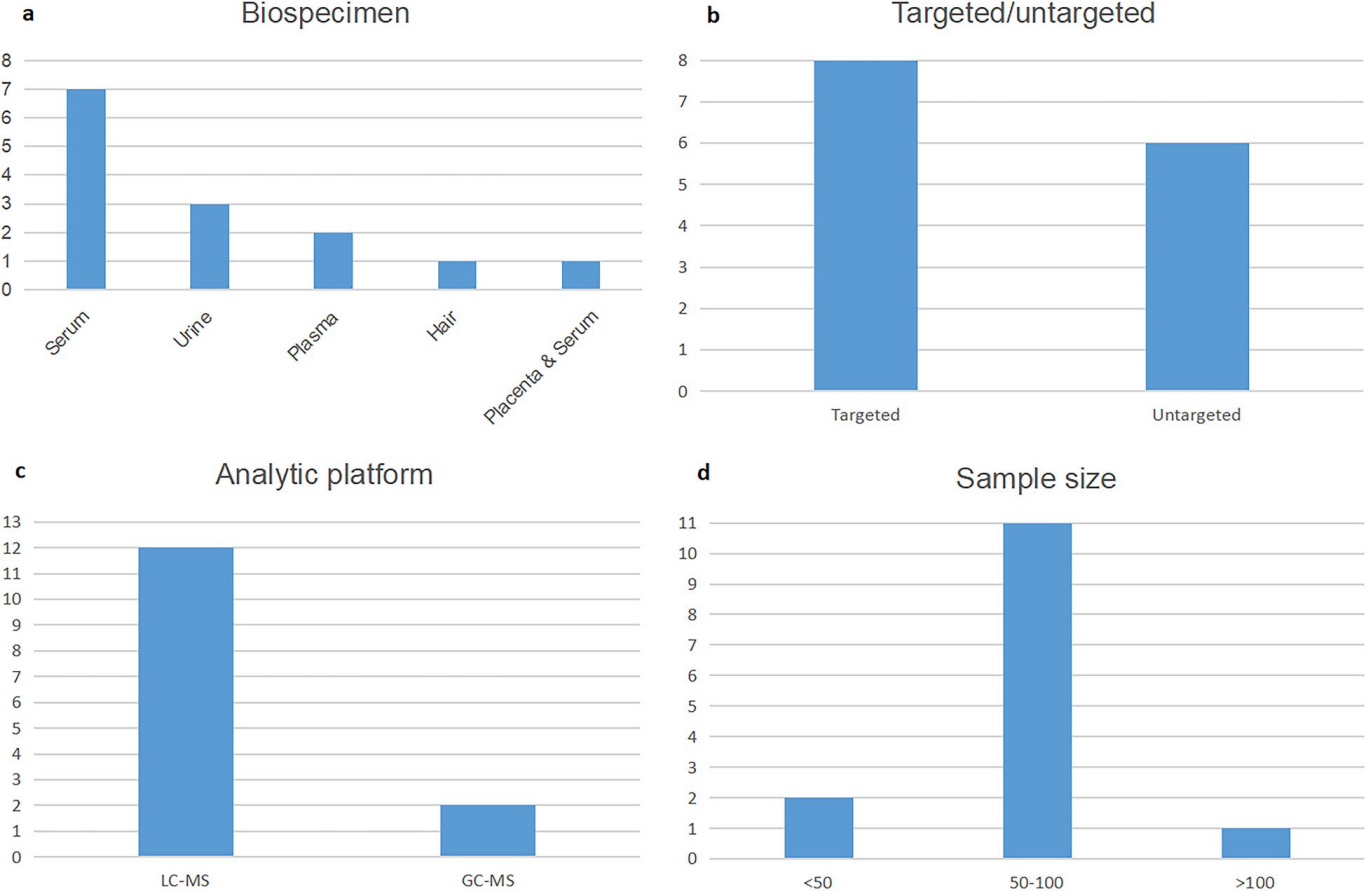
Numbers of the included studies according to biospecimen (**a**), targeted/untargeted (**b**), analytic platform (**c**), and sample size (**d**)

A total of 14 articles [9, 12–24] were included in this systematic review (Fig. 1). The characteristics of the 14 studies are presented in Table 1. Nine studies were performed with blood samples (serum and plasma), three with urine samples, one with hair samples, and one simultaneously collected placenta and serum (Fig. 2a). Besides, 8 metabolomics studies were targeted and 6 were untargeted (Fig. 2b). Twelve studies used liquid chromatography–mass spectrometry (LC–MS) and the others used gas chromatography–mass spectrometry (GC–MS) (Fig. 2c). As for sample sizes, the majority of the studies ranged from 50 to 100 subjects (Fig. 2d).

**Table 1:** Characteristics of the 14 Included Studies

First Author (Year)	Country	Outcome	Case	Control	Bio specimen	Sampling time	Analytic platform	Targeted/untargeted	Up-regulated	Down-regulated
Yifan He (2022) [12]	China	ICP	93	75	Serum	Not mentioned	UPLC-QTOF-MS/MS	Targeted	Glycocholic acid; Taurocholic acid; Glycochenodeoxycholic acid; Taurochenodeoxycholic acid; Glycoursodeoxycholic acid; Tauroursodeoxycholic acid; Glycodeoxycholic acid; Taurodeoxycholic acid; Hyocholic acid; Glycohyocholic acid; Taurohyocholic acid; Glycolithocholic acid; Taurolithocholic acid; Glycohyodeoxycholic acid; Taurohyodeoxychlic acid; ω-Muricholic acid; Tauro-ω- muricholic acid; Tauro-α- muricholic acid; Gdi-1; Tdi-1; Tri-1; Tri-3; Gtri-1; Gtri-3; Gtri-4; Gtri-5; Gtri-6; Gtri-7; Gtri-8; Ttri-1; Ttri-2; Ttri-3; Ttri-4; Ttri-5; Di-S-1; Di-S-2; Di-S-3; Di-S-4; Di-S-5; Gdi-S-1; Gdi-S-2	Chenodeoxycholic acid; Deoxycholic acid; Lithocholic acid; Di-1; Di-2; Tri-5
Ruirui Dong (2021) [13]	China	ICP	10	10	Placenta	After caesarian section	LC–MS/MS	Untargeted	Glyceraldehyde; l-Proline; Glycocholic acid; l-Palmitoylcarnitine	d-Glucuronic acid; Thyroxine; Xanthurenic acid; Levulinic acid; n-Oleoylethanolamine
			40/24/20	40/40/33	Serum	During the third/first/second trimester		–	l-Palmitoylcarnitine	None
Jamie V.de Seymour (2018) [14]	China	ICP	38	46	Hair	17-41 W	GC–MS	Untargeted	None	None
Yuchao Li (2018) [15]	China	Mild ICP Severe ICP	29(11 + 18)	22	Urine	At the first visit for the definite diagnosis	HPLC–MS/MS	Targeted	Di-GBA-S-3; Glycocholic acid-3S; Di-TBA-S-3; Di-TBA-S-2; Taurocholic acid-3S; Cholic acid-3S; Taurolithocholic acid-3S	None
Li Ma (2017) [16]	China	ICP	30	30	Urine	At the third-trimester (≥ 28 W)	HPLC/Q–TOF–MS	Untargeted	LysoSM(d18:1); 17-Hydroxy-E4-neuroprostane; Dynorphin A (6–8); Varanic acid; MG(22:5(7Z,10Z,13Z,16Z,19Z)/0:0/0:0); LysoPE(22:5(7Z,10Z,13Z,16Z,19Z)/0:0); 5b-Cyprinol sulfate; 3-Oxooctadecanoic acid; Testosterone glucuronide; Phosphorylcholine; Xanthine; Dodecanedioic acid; 18-Oxocortisol; Tauromuricholic acid; Glycocholic acid; Pyridinoline; Chenodeoxyglycocholic acid; n-Ribosylhistidine; Chenodeoxycholic acid-3S; 1-Methylguanosine; Glycochenodeoxycholate-3S; Taurohyocholate; (Z)-Narceine imide; 11-Oxo-androsterone glucuronide; 2-Deoxypentonic acid; Oxidized glutathione; Estrone glucuronide; Estriol-3-glucuronide	l-Homocysteine sulfonic acid; Galactonic acid; Isocitric acid; Cortolone-3-glucuronide
Yue Cui (2018) [9]	China	ICP	42	55	Serum	At the first time visit to take the confirmation diagnosis	UPLC-Triple TOF–MS/MS	Targeted	Tauroursodeoxycholic acid; Taurohyodeoxychlic acid; Taurochenodeoxycholic acid; Taurodeoxycholic acid; Glycohyodeoxycholic acid; Glycochenodeoxycholic acid; Glycodeoxycholic acid; ω-Muricholic acid; α-Muricholic acid; β-Muricholic acid; Hyocholic acid; Tauro-ω- muricholic acid; Tauro-α-muricholic acid; Taurohyocholic acid; Taurocholic acid; Glycohyocholic acid; Glycocholic acid; Taurolithocholic acid; Glycolithocholic acid; Tdi-1; Gdi-1; Tri-1; Tri-2; Tri-3; Tri-4; Ttri-1; Ttri-2; Ttri-3; Ttri-4; Ttri-5; Gtri-1; Gtri-2; Gtri-3; Gtri-4; Gtri-5; Gtri-6; Gtri-7; Gtri-8; Di-S-1; Di-S-2; Di-S-3; Di-S-4; Di-S-5; Gdi-S-1; Gdi-S-2; Gdi-S-3; Gdi-S-4; Gdi-S-5; Gtri-S-1	Di-2
X Sun (2021) [17]	China	Mild ICP Severe ICP	60(30 + 30)	30	Plasma	On the second day after the initial diagnosis of ICP	LC–MS/MS	Untargeted	SM(d34:0); SM(d18:0/16:0); SM(d42:1); SM(d22:0/18:1); Cer(d18:0/24:1); SM(d20:0/16:0); SM(d18:1/24:1); SM(d24:0/18:2); PC(32:0); PC(40:5); PI(16:0/22:6)	TG(18:3/18:2/18:2); TG(18:2/18:2/18:2); SM(d22:1/19:1); SM(d41:2); Cer(d18:1/23:0); SM(d39:1); SM(d41:1); SM(d22:1/18:0); PS(36:1); PC(17:0/18:2); PC(35:2); PS(41:5); PE(36:2p); Cer(d18:2/24:0); Cer(d18:1/22:0); Cer(d18:1/24:0); SM(d22:0/18:2); TG(16:0/18:1/18:3); DG(34:3e)
Qihong Zheng (2021) [18]	China	Mild ICP Severe ICP	32(14 + 18)	28	Plasma	Not mentioned	HPLC–MS/MS	Targeted	Norcholic acid; Glycochenodeoxycholic acid; Glycocholic acid; Taurocholic acid; Hyocholic acid; Glycohyocholic acid; Taurochenodeoxycholic acid; Taurohyocholic acid; Taurolithocholic acid-3S; Glycoursodeoxycholic acid	3-β-Cholic acid
Jianbo Chen (2013) [19]	China	Mild ICP	28	35	Serum	In the last trimester of pregnancy	HPLC–MS/MS	Targeted	Glycocholic acid; Glycochenodeoxycholic acid; Glycoodeoxycholic acid; Taurocholic acid; Taurodeoxycholic acid; Tauroursodeoxycholic acid	None
		Severe ICP	33	35					Cholic acid; Glycocholic acid; Glycochenodeoxycholic acid; Glycodeoxycholic acid; Taurocholic acid; Taurochenodeoxycholic acid; Taurodeoxycholic acid; Tauroursodeoxycholic acid	None
Lian Ye (2007) [20]	China	ICP	43	53	Serum	Not mentioned	HPLC–MS/MS	Targeted	Glycocholic acid; Glycochenodeoxycholic acid; Taurocholic acid; Tauroursodeoxycholic acid	Taurochenodeoxycholic acid
Antonín Pařízek (2016) [21]	Czech Republic	ICP	15	17	Serum	Not mentioned	GC–MS	Untargeted	16α-Hydroxyestrone; Conjugated epipregnanolone (3β,5β-THP); Conjugated 5α-pregnane-3α,20α-diol; Androsterone (3α,5α-THA) sulfate; Epiandrosterone (3β,5α-THA) sulfate; Epietiocholanolone (3β,5β-THA) sulfate; Conjugated 5α-androstane-3α,17β-diol; 3α,5α,20α-PD/3α,5α-THA, conjugates; 3β,5α,20α-PD/3β,5α-THA, conjugates; 3α,5β,20α-PD/3α,5β-THA, conjugates; 3β,5β,20α-PD/3β,5β-THA, conjugates	None
Guo-Hua Li (2020) [22]	China	ICP	15	15	Serum	38 W(case) 33.4 W(control)	LC–MS	Untargeted	Tauroursodeoxycholic acid; Glycolithocholic acid; Taurochenodeoxycholate; Cholic acid; Glycodeoxycholic acid; Glycochenodeoxycholate; Glycocholic acid; Chenodeoxycholate; Pregnenolone sulfate; Progesterone; l-Palmitoylcarnitine; Creatinine; 1-Aminocyclopropanecarboxylic acid; 1-Palmitoyl Lysophosphatidic Acid; 3-Methoxy-4-Hydroxyphenylglycol Sulfate; 2-Hydroxy-3-methylbutyric acid; DL-3-Phenyllactic acid; Alpha-N-Phenylacetyl-l-glutamine; N6-methyladenosine; 1-Methylnicotinamide; Succinate; 1-Oleoyl-L-alpha-lysophosphatidic acid; Ramipril; S-Methyl-5'-thioadenosine; Adenine; l-Threonine; l-Glutamine; l-Pyroglutamic acid; d-Proline; l-Tyrosine; Gly-Glu; Sphingosine; N6,N6,N6-Trimethyl-l-lysine; N6-Methyl-l-lysine; Urea; Adynerin	1-Myristoyl-sn-glycero-3-phosphocholine; n-(omega)-Hydroxyarginine; Inosine; Betaine; Phe-Trp; Phe-Phe; His-Ala; Pro-Arg; His-Glu; Ser-Arg; His-Ser; His-Gly; Pro-Val; Phe-Gly; Phe-Ile; His-Gln; Lys-Ser; Phe-Thr; Phe-Pro; His-Thr; Val-His; His-Ile
Xiao Chen (2019) [23]	China	ICP	33	35	Urine	> 28 W	UPLC-QTOF-MS/MS	Targeted	Tauroursodeoxycholic acid; Taurochenodeoxycholic acid; Glycoursodeoxycholic acid; Glycohyodeoxycholic acid; Tauro-ω- muricholic acid; Tauro-α-muricholic acid; Taurohyocholic acid; Taurocholic acid; Glycocholic acid; Taurolithocholic acid-S; Tdi-1; Tdi-2; Tdi-3; Gdi-1; Ttri-1; Ttri-2; Ttri-3; Ttri-4; Ttri-5; Gtri-2; Di-S-3; Di-S-4; Di-S-5; Di-S-6; Di-S-7; Di-S-8; Tdi-S-1; Tdi-S-2; Tdi-S-3; Tdi-S-4; Tdi-S-5; Tdi-S-6; Gdi-S-1; Gdi-S-2; Gdi-S-3; Gdi-S-4; Ttri-S-1; Ttri-S-2; Gtri-S-1; Gtri-S-2; Mono-S; Gmono-S-1; Gmono-S-2; Gmono-S-3; Gmono-S-4	None
Rachel M. Tribe (2010) [24]	UK	ICP	63	26	Serum	16–40 W	HPLC–MS	Targeted	Cholic acid; Taurocholic acid; Taurochenodeoxycholic acid; Taurolithocholic acid; Tauroursodeoxycholic acid; Glycocholic acid; Glycochenodeoxycholic acid	None

The prefix “G-” or “T-”mean the bile acids exist in glycine- or taurine-conjugated form, respectively. The prefix “mono-”, “di-”, or “tri-” mean the bile acids may have one, two, or three -OHs, respectively. The prefix “S-” means the BAs are sulfated

*GBA* Glycine bile acid; *TBA* Taurine bile acid

### Analysis of metabolic biomarkers of ICP

Of the 14 studies included, only one metabolomics article used hair and did not identify statistically meaningful metabolites [14]. The other 13 found 212 metabolic biomarkers that were significantly associated with ICP. In addition, since most studies focused on the metabolic profile of bile acids, the high-frequency biomarkers (reported in ≥ 4 studies) involved were limited and were all bile acids. Glycocholic acid (GCA) was totally reported ten times, which was the most frequently reported metabolite. Interestingly, except for taurochenodeoxycholic acid (TCDCA), which showed a down-regulated trend in one study [20], all other significant bile acids showed an up-regulated trend in ICP (Table 2).

**Table 2:** High-frequency metabolic biomarkers of ICP

Bile acids	Frequency	Bio-specimen	References
GCA (glycocholic acid)	10	Serum	[9, 12, 19, 20, 22, 24]
		Plasma	[18]
		Urine	[16, 23]
		Placenta & Serum	[13]
TCDCA (taurochenodeoxycholic acid)	8	Serum	[9, 12, 19, 22, 24] [20]^a^
		Plasma	[18]
		Urine	[23]
TCA (taurocholic acid)	7	Serum	[9, 12, 19, 20, 24]
		Plasma	[18]
		Urine	[23]
TUDCA (tauroursodeoxycholic acid)	7	Serum	[9, 12, 19, 20, 22, 24]
		Urine	[23]
GCDCA (glycochenodeoxycholic acid)	7	Serum	[9, 12, 19, 20, 22, 24]
		Plasma	[18]
THCA (taurohyocholic acid)	5	Serum	[9, 12]
		Plasma	[18]
		Urine	[16, 23]
GDCA (glycodeoxycholic acid)	4	Serum	[9, 12, 19, 22]

^a^Down-regulated trend

### Analysis of metabolic pathways

**Fig. 3:**
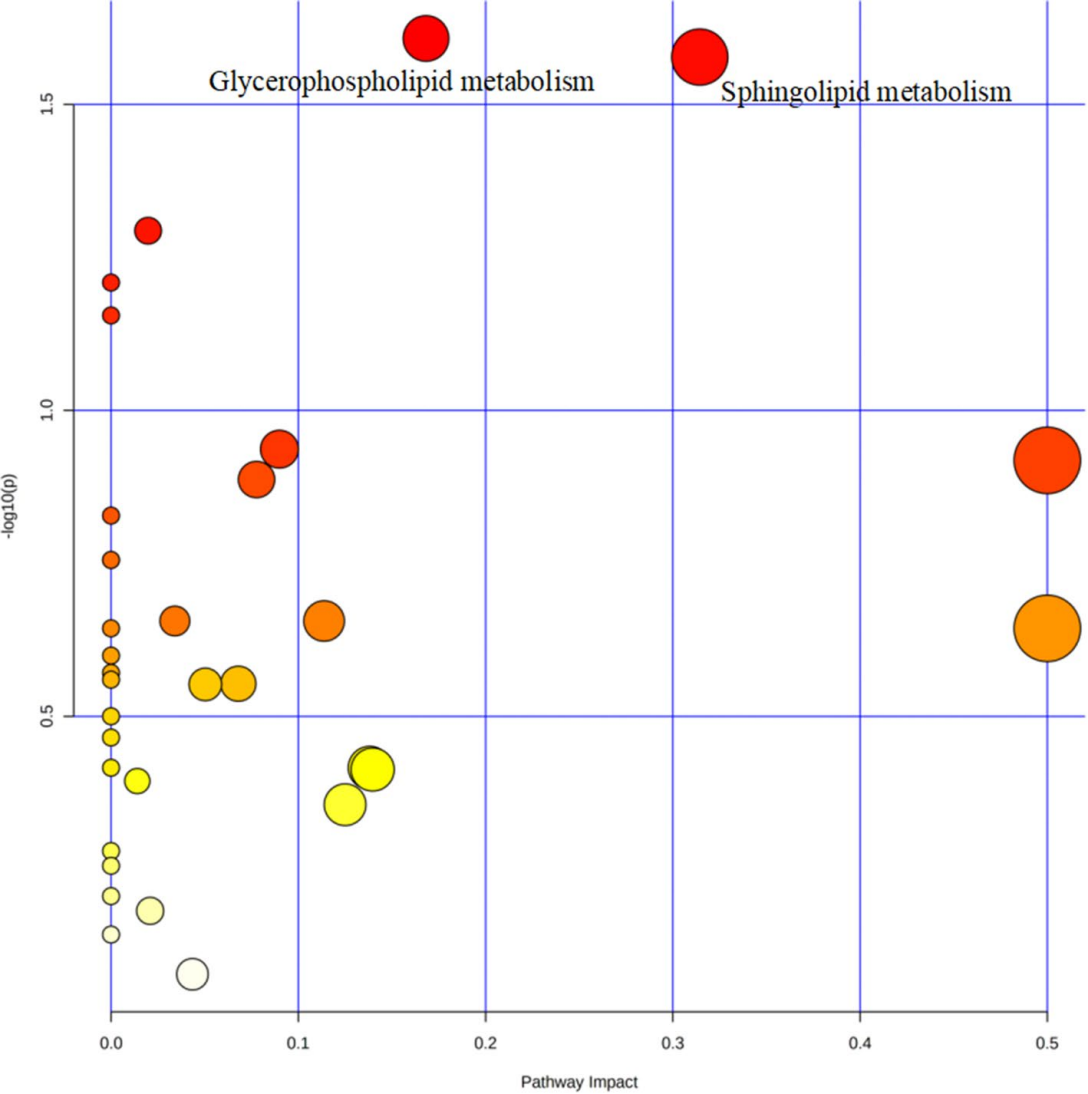
Overview of Pathway Analysis of ICP

To understand the metabolic pathways that these potential biomarkers are involved in, we imported all the reported metabolites except bile acids to MetaboAnalyst for pathway analysis, as bile acid metabolism is well-known to be related to ICP. As a result, 98 non-bile acid metabolites were finally selected for the enrichment analysis. Detailed information regarding the analysis result is shown in Table 3. Two pathways were significantly enriched at the significance level of 0.05, namely, glycerophospholipid metabolism and sphingolipid metabolism, which are both lipid metabolism-related pathways (Fig. 3). The glycerophospholipid metabolism pathway includes phosphatidylcholine (PC), phosphorylcholine, and phosphatidylserine (PS), and the sphingolipid metabolism pathway contains sphingomyelin (SM) and ceramide (Cer).

**Table 3:** Results of the Pathway Analysis

Pathway name	Raw *P* value	Impact
Glycerophospholipid metabolism	0.02467	0.16820
Sphingolipid metabolism	0.02646	0.31440
Purine metabolism	0.05086	0.01979
Aminoacyl-tRNA biosynthesis	0.06181	0.00000
Arginine biosynthesis	0.06995	0.00000
Arginine and proline metabolism	0.11571	0.08992
Phenylalanine, tyrosine and tryptophan biosynthesis	0.12069	0.50000
Citrate cycle (TCA cycle)	0.12970	0.07771
Linoleic acid metabolism	0.14856	0.00000
D-Glutamine and D-glutamate metabolism	0.17556	0.00000
Nitrogen metabolism	0.17556	0.00000
Glutathione metabolism	0.22087	0.03407
Alanine, aspartate and glutamate metabolism	0.22087	0.11378
Valine, leucine and isoleucine biosynthesis	0.22708	0.00000
Ascorbate and aldarate metabolism	0.22708	0.50000
Ubiquinone and other terpenoid–quinone biosynthesis	0.25164	0.00000
Glyoxylate and dicarboxylate metabolism	0.26846	0.00000
Phenylalanine metabolism	0.27544	0.00000
Steroid hormone biosynthesis	0.27967	0.06797
Glycine, serine and threonine metabolism	0.28040	0.05034
Arachidonic acid metabolism	0.31612	0.00000
alpha-Linolenic acid metabolism	0.34246	0.00000
Butanoate metabolism	0.38373	0.00000
Nicotinate and nicotinamide metabolism	0.38373	0.13816
Tyrosine metabolism	0.38627	0.13972
Glycerolipid metabolism	0.40341	0.01402
Pentose and glucuronate interconversions	0.44092	0.12500
Propanoate metabolism	0.52489	0.00000
Lysine degradation	0.55491	0.00000
Inositol phosphate metabolism	0.62205	0.00000
Cysteine and methionine metabolism	0.65746	0.02089
Pyrimidine metabolism	0.71880	0.00000
Drug metabolism—cytochrome P450	0.83451	0.04348

### Predictive and diagnostic potential of metabolite markers for discriminating ICP

Five studies [9, 13, 15, 16, 18] evaluated the potential of metabolic biomarkers or biomarker panels to predict and diagnose ICP (Table 4). These studies all calculated the area under the receiver operating curve (AUC) of single metabolites, resulting AUC values ranging from 0.642 to 1.000. Besides, Cui et al. [9] and Zheng et al. [18] both used bile acids panels to predict or diagnose ICP. Adding Complementary other biomarkers to bile acids was also shown to be effective in Dong et al. and Ma et al.’s studies [13, 16]. In addition, Dong et al. [13] indicated that metabolites at different stages of pregnancy had different predictive and diagnostic abilities.

**Table 4:** Potential of metabolic markers for the prediction or diagnosis of ICP

References	Biospecimen	Potential Biomarker(s) or Biomarker Panel	AUC	Sensitivity	Specificity
Ruirui Dong (2021) [13]	Serum	l-Palmitoylcarnitine	0.896(the third trimester)	–	–
			0.657(the first trimester)	–	–
			0.727(the second trimester)	–	–
		Glycocholic acid	0.985(the third trimester)	–	–
			0.686(the first trimester)	–	–
			0.670(the second trimester)	–	–
		l-Palmitoylcarnitine + Glycocholic acid + ACOX1^a^	0.993(the third trimester)	–	–
			0.891(the first trimester)	–	–
			0.932(the second trimester)	–	–
Yuchao Li (2018) [15]	Urine	Di-GBA-S-3	0.975	0.966	0.909
		Glycocholic acid-3S	0.997	1.000	0.955
		Di-TBA-S-3	0.929	0.862	1.000
		Di-TBA-S-2	0.983	0.966	1.000
		Taurocholic acid-3S	0.995	1.000	0.955
		Cholic acid-3S	0.873	0.793	0.955
		Taurolithocholic acid-3S	0.828	0.690	0.909
Li Ma (2017) [16]	Urine	32 differential metabolites	0.642–0.918	–	–
		MG (22:5) + LysoPE (22:5) + L-Homocysteine sulfonic acid + Glycocholic acid + Chenodeoxycholic acid-3S	0.988	0.900	0.933
Yue Cui (2018) [9]	Serum	Gtri-8	0.931	0.929	0.873
		Taurochenodeoxycholic acid	0.946	0.952	0.855
		Ttri-5	0.940	0.833	0.909
		Glycocholic acid	0.957	0.929	0.891
		Glycochenodeoxycholic acid	0.917	0.881	0.891
		Gtri-3	0.938	0.905	0.891
		Tauro-ω-muricholic acid	0.901	0.762	0.964
		Taurocholic acid	0.946	0.905	0.891
		Gtri-7	0.924	0.810	0.946
		α-Muricholic acid	0.876	0.810	0.891
		Gtri-6	0.950	0.905	0.873
		Taurocholic acid + α-Muricholic acid + Gtri-8	0.996	0.976	0.964
Qihong Zheng (2021) [18]	Plasma	Norcholic acid	0.900	0.781	0.929
		Glycocholic acid	0.994	0.969	1.000
		Glycochenodeoxycholic acid	0.977	0.938	0.929
		Glycohyocholic acid	0.998	1.000	0.964
		Glycoursodeoxycholic acid	0.894	0.813	0.857
		Hyocholic acid	0.819	0.594	1.000
		Taurocholic acid	1.000	1.000	1.000
		Taurochenodeoxycholic acid	1.000	1.000	1.000
		Taurohyocholic acid	0.992	0.938	1.000
		Above 9 metabolites	1.000	1.000	1.000

The prefix “G-” or “T-” mean the bile acids exist in glycine- or taurine-conjugated form, respectively. The prefix “di-”, or “tri-” mean the bile acids may have two, or three -OHs, respectively. The prefix “S-” means the BAs are sulfated

*GBA* Glycine bile acid; *TBA* Taurine bile acid

^a^ACOX1, Acyl-CoA oxidase 1 (different protein)

## Discussion

In this study, 14 metabolomics studies on ICP were comprehensively reviewed and analyzed. To identify valuable metabolic biomarkers, seven high-frequency metabolites (reported in ≥ 4 studies) were listed. Pathway analysis results indicated two metabolic pathways involved in ICP and suggested a series of metabolic dysregulations in ICP patients.

### Bile acid metabolism and ICP

Several bile acids were repeatedly identified across these studies. Bile acids, the main component of bile, are steroidal C24 carboxylic acids formed from cholesterol metabolism [25]. Based on synthetic pathways, bile acids can be classified into primary bile acids (i.e., cholic acid and chenodeoxycholic acid), secondary bile acids, and tertiary bile acids [26]. Bile acids can also be divided into 2 categories: free and conjugated bile acids (conjugated with taurine or glycine) [27].

Under normal conditions, bile acids synthesized in the liver are secreted into the bile, stored in the gallbladder, reabsorbed in the intestine, and transported back to the liver. This process is known as the reabsorption of bile acids, the so-called enterohepatic circulation [28]. Therefore, almost all bile acids are sustained in the enterohepatic system and maintained “sequestered”. In ICP, due to disruption of bile acid transport, bile acids are accumulated in liver cells, increasing their flow to the maternal systemic blood circulation and elevating circulating bile acid concentration [29]. In our results, high-frequency biomarkers were almost bile acids that showed an up-regulated trend in ICP. Only 1 study suggested that the concentration of TCDCA in ICP was significantly lower than in healthy controls [20]; however, the article did not mention the ICP diagnostic criteria and sampling time, and used a new quantitative method at that time, which may lead to the inconsistence. In addition, it is not difficult to find that these high-frequency bile acids were all conjugated bile acids, including four taurine conjugated and three glycine conjugated. Conjugated bile acids are more hydrophilic and less toxic than free bile acids [30]. Extravasation of bile acids and elevation of conjugated bile acids may be maternal adaptive mechanisms during cholestasis to reduce bile acids toxicity to the liver [31]. Studies have also demonstrated that serum conjugated bile acids significantly increased in various hepatopathies [32–34]. In ICP, especially severe ICP, fetal complications are more closely associated with serum TBA levels. It has been reported that for every 1 μmol/L increase in serum TBA, the incidence of fetal complications (including preterm delivery, asphyxial events, and meconium staining) increases by 1–2% [35]. Therefore, the disorders of bile acid metabolism and variation in bile acid profile require further exploration of methods to reduce serum TBA concentration and improve pregnant outcomes of ICP patients.

Though serum TBA concentration has been the most commonly used criterion for the diagnosis of ICP, metabolomics may provide more specific disease information. Single [9, 13, 15, 16, 18] or panels [9, 18] of bile acids, or combining bile acids with other biomarkers [13, 16], all have the potential to effectively predict and diagnose ICP. However, the related studies generally had a small sample size and limited validation. Further studies, such as independent external cohorts with a large sample size, are needed for obtaining reliable conclusions. In addition, Dong and coworkers [13] suggested that biomarkers’ discriminating ability differed according to stages of pregnancy. Therefore, clinical applications of bile acids panels require further exploration and optimization.

### Lipid metabolism and ICP

Although the changes in bile acid metabolites were the most significant, lipid alterations would also help in the understanding of ICP pathogenesis. As basic components of cellular membranes, lipids are crucial for maintaining cellular structure, function, signaling, and energy storage [36]. Therefore, disruptions of lipid metabolism and transport exert a certain effect on human disease. In a study with a sample size of 63 ICP patients [37], results showed that ICP was associated with an abnormal lipid profile: low-density lipoprotein cholesterol, high-density lipoprotein cholesterol, total cholesterol, and other plasma lipid concentrations were all significantly changed. Similarly, a lipidomics study [38] observed that multiple lipid components had been altered in mice with alpha-naphthyl isothiocyanate-induced intrahepatic cholestasis, suggesting that lipid metabolism disorder might be the underlying pathogenesis of intrahepatic cholestasis.

PC is a structural lipid and shows a downward trend in ICP patients [17]. Disorders in the secretion of PC into bile, as well as a significant decrease in phospholipid concentrations in bile, can lead to cholangiocyte luminal membrane injury and biliary lesions that cause cholestasis [39]. PS is also a glycerophospholipid, and its metabolism is associated with ATP8B1, which acts as the flippase for PS [40]. Studies have demonstrated that mutations in ATP8B1 could cause cholestatic disease [41] and increase the risk of ICP [42]. The pathogenic mechanism lies in that the mutations in ATP8B1 could lead to the loss of phospholipid asymmetry and subsequently undermined bile salt transport [43].

SM is a type of sphingolipid found in animal cell membranes and usually consists of phosphorylcholine and Cer. Serving as modulators of liver regeneration, sphingolipids and their enzymes play a key role in repairing liver injury [44]. Cer is one of the hydrolysis byproducts of SM by the enzyme sphingomyelinase. A study [45] focusing on the role of sphingolipids in the pathogenesis of intrahepatic cholestasis found that the levels of Cers were significantly elevated in the ICP group, and Cers could be potentially used as early biomarkers of ICP. An animal experiment [46] noticed that Cer/SM imbalance would promote lipid metabolism disorder and apoptosis and gradually cause liver injury. Thus, there is a strong relationship between SM and Cer, and the changes in their levels can reflect the health status of human live, which can be regarded as a potential therapeutic target of ICP.

### Limitations of current metabolomics studies on ICP

Several limitations of the existing metabolomics studies on ICP should be noted. First, the majority of subjects were recruited in China, which may result in the limitation on study population. Moreover, most researchers [9, 12, 15, 18–20, 23, 24] focused on bile acid metabolism. Second, many studies possessed relatively small sample sizes, which may influence statistical power and the credibility of their research results. Third, when researchers used biofluids as samples, profiling of metabolites may be greatly dynamic and influenced by multiple factors including diet, immune status, lifestyle, and so on [47]. For example, bile acid profiles often varied from fasting to non-fasting conditions. In addition, the metabolomics on ICP is still in the preliminary stage of development. Before translating the results into clinical practice, more independent validations are needed. Sufficient external cohorts or animal/cell experiments are necessary to verify, complement and deepen current findings. Finally, the integration of metabolomics with other omics (e.g., genomics, transcriptomics, and proteomics) may help researchers to obtain a comprehensive understanding of the complexity of ICP.

## Conclusions

To sum up, this study conducted a systematic review and analysis of metabolomics research on different aspects of ICP. Except for bile acid metabolism, glycerophospholipid metabolism and sphingolipid metabolism were suggested to be changed in patients with ICP. The reported metabolic biomarkers suggested potential applications of metabolomics in the clinical prediction and diagnosis of ICP. Therefore, more comprehensive and improved metabolomic studies should be encouraged to provide more valuable information for the exploration and understanding of ICP.

## Data Availability

Not applicable.

## Abbreviations

ICP: Intrahepatic cholestasis of pregnancy
TBA: Total bile acid
LC–MS: Liquid Chromatography–mass spectrometry
GC–MS: Gas chromatography–mass spectrometry
GCA: Glycocholic acid
TCDCA: Taurochenodeoxycholic acid
PC: Phosphatidylcholine
PS: Phosphatidylserine
SM: Sphingomyelin
Cer: Ceramide
AUC: Area under the receiver operating curve
